# Supplementary material for: Maternal COVID-19 infection and associated factors: A cross-sectional study
Source: PLoS One. 2023 Mar 15;18(3):e0281435. doi: 10.1371/journal.pone.0281435 (PMC10016676; doi:10.1371/journal.pone.0281435)
Supplement: Attachment — Submitted filename: Response to Reviewers_131222.docx [file pone.0281435.s001.docx]

6^th^ December, 2022.

The Academic Editor,

PLOS ONE Journal,

Dear Dr Gbenga Olorunfemi,

# **RE:** Manuscript ID: PONE-D-22-21448__ Title: Maternal COVID-19 Infection and Associated Factors: A Cross-Sectional Study

We are sincerely greatful that you invited us to submit a revised version of the manuscript that addresses the points raised during the review process. We took all the points and comments raised with the seriousness they deserve as we believe this would help in improving the manuscript achieve the publication criteria. Below in the table is the point-by point rebuttal. Thank you once again for taking your time to review our manuscript.

Yours sincerely,

Mwansa Ketty Lubeya

Corresponding author

| **Reviewers’ Comments** | **Changes made/response/clarification** | **Line number in revised manuscript with track changes** |
| --- | --- | --- |
| **Academic Editor’s comments** |  |  |
| 1. May you ensure that you utilize the appropriate sign symbol of PLOS ONE in your Tables. Please ensure that your manuscript meets PLOS ONE's style requirements, including those for file naming. The PLOS ONE style templates | We appreciate this guidance. We have since adjusted our Tables to reflect plos one guidelines. And also the manuscript has been revised to follow the PLOS ONE journal requirements by the links that were given for style, file naming etc |  |
| 2. You indicated that you had ethical approval for your study. Please clarify whether minors (participants under the age of 18 years) were included in this study. | Thank you for this comment. This study did not recruit any minors therefore to clarify this point we have added the following:  “All women older than 18 years who presented……..” | Line 142 |
| 3. Please remove any funding-related text from the manuscript and let us know how you would like to update your Funding Statement. Currently, your Funding Statement reads as follows: “This study did not receive any funding” | Thank you for this guidance. We have edited and removed all text that was put in the acknowledgement to imply as though this study did receive any funding when in fact not. Therefore, we are not updating our funding statement as these were mere acknowledgements as this study did not receive any funding. | Line 361-366 |
| 4. Thank you for stating the following financial disclosure:   “This study did not receive any funding”  At this time, please address the following queries:   a) Please clarify the sources of funding (financial or material support) for your study. List the grants or organizations that supported your study, including funding received from your institution. b) State what role the funders took in the study. If the funders had no role in your study, please state: “The funders had no role in study design, data collection and analysis, decision to publish, or preparation of the manuscript.” c) If any authors received a salary from any of your funders, please state which authors and which funders. d) If you did not receive any funding for this study, please state: “The authors received no specific funding for this work.” | Thank you for comment on financial disclosure and sincere apologies for the unclear statements earlier. We share address the sub-querries point by point:  4a) There were no sources of funding for this research in particular. The authors just put their resources together to ensure that the study was done.  4b) Since they were no funders we believe this point is not applicable  4c) No authors received any salary to have this study done  4d) “The authors received no specific funding for this work.” We have since maintained this statement and now hope that our financial disclosure is clear. | Line 354 |
| 5. Thank you for stating the following in your Competing Interests section:   “No authors have a competing interest”  Please complete your Competing Interests on the online submission form to state any Competing Interests. If you have no competing interests, please state "The authors have declared that no competing interests exist." | Thank you for this comment. The authors have no competing interests therefore we have edited the statement as guided to read: "The authors have declared that no competing interests exist." | Line 357 |
| 6. We note that you have indicated that data from this study are available upon request. PLOS only allows data to be available upon request if there are legal or ethical restrictions on sharing data publicly. a) If there are ethical or legal restrictions on sharing a de-identified data set, please explain them in detail (e.g., data contain potentially sensitive information, data are owned by a third-party organization, etc.) and who has imposed them. b) b) If there are no restrictions, please upload the minimal anonymized data set necessary to replicate your study findings as either Supporting Information files or to a stable, public repository and provide us with the relevant URLs, DOIs, or accession numbers. | Many thanks for this comment and guidance, we have since revised and addressed this comment point by point as follows:  6a) There are no ethical or legal restrictions on sharing a de-identfied data set  6b) We have upload the minimal anonymized data set necessary to replicate our study findings and can be found at the following link: <https://doi.org/10.6084/m9.figshare.21671588> and we have also updated in the manuscript | Line 375-376 |
| 7. You may report the univariable regression results alongside the multivariable if it is relevant to your presentation | Thank you for the guidance. We do think that it is sufficient to remain in the table and only report the multivariable results. We have since dropped this part from the text in the results.  We hope this is now acceptable | Line 247-253 |
| **Reviewer 1** |  |  |
| 1. Abstract and Methodology: The authors should clarify how the test was done either routinely or voluntarily? What is the testing protocols in the study hospitals? How long does the test take to come out? Who paid for the COVID-19 test? How was the sampling technique achieved in the study? | Thank you for the queries, we have since clarified by the responses below as well as in the manuscript according:  -COVID-19 tests were routinely done all the women who were seeking services during antenatal, intrapartum, and postnatal care.  - All women who visited the hospital were tested for COVID-19 at the entry points which included antenatal clinic, labour ward, emergency triage unit, and emergency gynae ward. The swabbing was done by the attending medical doctor, or nurse, or environmental health officer. The Rapid Diagnostic Test (RDT) was read immediately, while the PCR was placed in a cool-box which was sent to the laboratory within an hour of collection.  - The PCR test results took between 24-48 hours to come out  - The test was free of charge as the government facilitated the provision of nasopharyngeal swabs and laboratory testing in these two facilities through various donations it received.  - Total enumeration was done for women who presented between March – July 2021, subject to eligibility criteria being met and consent gotten. | Lines 149-154 |
| 2. Results: The authors should begin by stating how many participants were assessed for eligibility and how many were recruited, excluded and reason for exclusion. | We have added the following sentence in the results section for more clarity  “We screened 385 participants, with 33 not meeting the eligibility criteria.” | Line 225-226 |
| **Reviewer 2** |  |  |
| 1. Introduction: In the introduction section paragraph 2. You said “Since the early days of the pandemic, there has been conflicting literature regarding the risk of COVID-19 in pregnant women [5-7]”. What does it mean? I think all your cited article showed that pregnant women are more venerable for COVID-19 than the general population. What was the controversy idea? | Many thanks for this querry. We would like to clarify as follows and elobarate further on the studies that were cited. We hope this addresses the querry now.  “Since the early days of the pandemic, there has been conflicting literature regarding the risk of COVID-19 in pregnant women. While some studies done in China and in Senegal do not suggest an increased risk of severe disease among pregnant women compared to the general population [5,6], another study in France suggested increased respiratory morbidity related to COVID-19 infection [7].” | Line 88-91 |
| 2. Sample size calculation: Your sample is not correct. Based on your assumption your sample size 384 (before adding non response rate). After considering 10% of non-response rate the final sample size should be 422. Please check it. | Thank you for this question. However, the sample size before non-response rate was indicated to be 347 at the 5% level of significance, 95% confidence intervals, and 5% margin of error. Therefore, accounting for 10% non-response rate gave us a sample size of 385 | Line 143-144 |
| 3. Enrolment procedure: As you said in WNH-UTH and LMUTH on average, 9000 and 8000 pregnant women seen annually, also your study period was March and July 2021 (almost 5 months). Accordingly, approximately around 7000 pregnant women visited this two hospitals with in your study period, On the other hand your sample size is 385. How can you draw your sample? Please say something about your sampling technique (how the actual sample was drawn? 4. How do you manage if the women visited the hospital more than once during the month? | We appreciate this query. We have elaborated this point further to help clarify:  During the study period there were an average of 1,600 visits by antenatal and postnatal women. New visits were about 100 women in a month, while revisits amounted to about 1,100 each month. Postnatal women were about 450 visits per month.  Total enumeration was done for women who presented between March – July 2021, subject to eligibility criteria being met and consent obtained.  Women who had already been enrolled in the study were not eligible for re-enrolment. | Lines 133- 136  Lines 157-160 |
| 5. What is the difference between pilot study and pretest? Which one is included in the analysis? | Thank you for this clarification. We used a pilot study and these participants were not included in final analysis. | Lines 169-172 |
| 6. Some results not give a total of 352 study participant. For example ANC visit, BMI, marital status, education, employed. | Thank you to the reviewer for this guidance.  We conducted a complete case analysis, as the missing data did not affect the estimates even after using multiple imputations technique for missing data.  Therefore the total for each variable are varied. To address this for more clarity we have added the actual “n” for each variable where data was missing and added explanation on the Table footnote as follows”  “all variables with missing values have been indicated with respective “n” used for complete case analysis”  We hope this is now ok | Table 1 |
| 7. Delete univariable analysis OR result from the text, it is enough to be presented in the table. | We appreciate this guidance and we have since dropped the univariable interpretation | Line 247- 253 |
| 8. What is the final result (multivariable logistic regression) of chronic hypertension? | We have added to the Table footnote the following:  “Chronic hypertension was dropped from final model due to colineality”  We hope this is ok | Table 2 |
| 9. Discussion: Further, studies have shown that people of colour are at an increased risk for COVID-19 infection which could explain the high prevalence we found at this setting. If there is any evidence cite? | We are grateful for this comment. We have added three new refences to cite this statement. | Line 282 |
